# The recovery of intestinal barrier function and changes in oral microbiota after radiation therapy injury

**DOI:** 10.3389/fcimb.2023.1288666

**Published:** 2024-02-07

**Authors:** Kun Wang, Jingjing Zhang, Yihao Zhang, Junze Xue, He Wang, Xiaojie Tan, Xuelong Jiao, Haitao Jiang

**Affiliations:** ^1^ Department of Gastrointestinal Surgery, Affiliated Hospital of Qingdao University, Qingdao, China; ^2^ Department of Pathology, Affiliated Hospital of Qingdao University, Qingdao, China

**Keywords:** 16s rRNA gene sequencing, rectal cancer radiotherapy, oral flora, intestinal mucosal barrier, rectal cancer

## Abstract

**Introduction:**

Colorectal cancer (CRC) is the third most common malignant tumor, and neoadjuvant chemo-radiotherapy is usually recommended for advanced stage colorectal cancer. Radiotherapy can cause damage to intestinal mucosal barrier, which may be related to perioperative complications. Intestinal microbiota is one of the constituents of the intestinal mucosal biological barrier, and literature reports that patients with CRC have changes in corresponding oral microbiota. This study aims to analyze the levels of immunoglobulin SIgA, inflammatory factors, lymphocyte subsets quantity, and proportion in surgical specimens of intestinal mucosa at different time intervals after radiotherapy, in order to seek investigation for the optimal surgical time after radiotherapy and to provide evidence for finding probiotics or immunomodulators through high-throughput sequencing of bacterial 16s rRNA in patients' saliva microbiota. Ultimately, this may provide new ideas for reducing perioperative complications caused by radiotherapy-induced intestinal damage.

**Methods:**

We selected intestinal mucosal tissue and saliva samples from over 40 patients in our center who did not undergo radiotherapy and underwent surgery at different time intervals after radiotherapy. Detection of SIgA was performed using ELISA assay. Western Blotting was used to detect IL-1β, IL-6, and IL-17 in the intestinal mucosal tissue. Flow cytometry was used to detect CD4 and CD8. And the microbial community changes in saliva samples were detected through 16s rRNA sequencing.

**Results:**

After radiotherapy, changes in SIgA, various cytokines, CD4CD8 lymphocyte subsets, and oral microbiota in the intestinal mucosal tissue of rectal cancer patients may occur. Over time, this change may gradually recover.

**Discussion:**

In colorectal cancer, oncological aspects often receive more attention, while studies focusing on the intestinal mucosal barrier are less common. This study aims to understand the repair mechanisms of the intestinal mucosal barrier and reduce complications arising from radiotherapy-induced damage. The relationship between oral microbiota and systemic diseases has gained interest in recent years. However, the literature on the oral microbiota after radiotherapy for rectal cancer remains scarce. This study addresses this gap by analysing changes in the salivary microbiota of rectal cancer patients before and after radiotherapy, shedding light on microbiota changes. It aims to lay the groundwork for identifying suitable probiotics or immunomodulators to alleviate perioperative complications and improve the prognosis of CRC.

## Introduction

The global consensus among experts is that preoperative radiotherapy is essential for advanced rectal cancer, given the rising incidence of rectal tumors. While radiotherapy offers benefits, it also poses adverse effects, including radiation-induced intestinal injury, a frequent complication following radiotherapy for pelvic, abdominal, or retroperitoneal tumors (Lin et al., 2023). Previous studies indicate that ionizing radiation, alterations in intestinal microbiota, and radiation-induced enteritis contribute to intestinal mucosal barrier damage (MacNaughton, 2000; Touchefeu et al., 2014; Fan et al., 2022). The intestinal barrier, apart from its roles in nutrient metabolism and absorption, includes the mucosal immune barrier formed by SIgA and intestinal associated lymphoid tissue, along with cytokines secreted by it (Sémont et al., 2006; Chen et al., 2021). This immune barrier prevents the entry of large molecules like bacteria and toxins into the bloodstream. Ionizing radiation can impair this barrier function, potentially leading to bacterial translocation, endotoxemia, and mucositis (Kwak et al., 2021). SIgA, the predominant antibody in mucosal secretions, maintains intestinal microbiota composition and homeostasis (Pietrzak et al., 2020). Several studies have linked intestinal mucosal barrier damage to various cytokines in intestinal mucosal tissue (Pietrzak et al., 2020; Dong et al., 2021). The intestinal epithelium and lamina propria, hosting a majority of CD8+T lymphocytes, are primary sites for T lymphocytes in the intestinal mucosa. Changes in intestinal immune function are reflected in the alterations of lymphocyte subtypes in the epithelium and lamina propria.

The human digestive tract houses symbiotic microbiota, ranging from the oral cavity to the rectum. These microorganisms play a crucial role in maintaining metabolic, immune, and endocrine homeostasis. The gut microbiota significantly influences the development, progression, metastasis, and therapeutic response of CRC (Garrett, 2015). The oral microbiome, a vital component of the oral cavity, helps prevent colonization by foreign bacteria, impacting overall health. Studies, including those by Flemer et al., have identified specific oral bacteria associated with CRC by analyzing microbial communities in oral, colonic mucosal tissues, and fecal samples from CRC patients (Flemer et al., 2018). Dong et al. showed that radiation therapy alters the salivary microbiota in mice, evident from comparisons between irradiated and non-irradiated mice (Dong et al., 2021).

This study assessed intestinal mucosal tissues from rectal cancer patients who underwent surgery without preoperative radiotherapy and those who had surgery 8-12 weeks post-radiotherapy. It measured levels of IL-1β, IL-6, IL-17, SIgA, CD4+, and CD8+ T lymphocytes in these samples. Additionally, saliva samples from over ten rectal cancer patients, with and without radiotherapy, were analyzed for bacterial 16s rRNA through high-throughput sequencing. Findings indicate that radiotherapy impairs the intestinal mucosal barrier, which partially recovers over time, but remains incomplete even after 8-12 weeks. Furthermore, radiotherapy induces alterations in oral microbial composition, suggesting that damage to the intestinal mucosal barrier might drive these changes.

## Materials and methods

### Ethics statement

Human bowel tissue samples were obtained from more than 40 rectal cancer patients who had undergone abdominal surgery at our centre within the previous 12 months by the same surgical team. The samples were divided into four groups: those who did not receive preoperative radiotherapy and those who underwent surgery 60 days ±2 days, 75 days ±2 days or 90 days ±2 days after completion of radiotherapy. In addition, 24 saliva samples were collected from rectal cancer patients, 11 of whom received preoperative radiotherapy and 13 of whom did not. Patients with a history of inflammatory bowel disease, chemotherapy, targeted therapy, other immunosuppressive drugs, or use of steroids and antibiotics within two weeks before surgery were excluded from the study. There were no significant differences in gender or age between the groups. All participants voluntarily signed the informed consent form. Sample collection was approved by the Ethics Committee of Qingdao University Affiliated Hospital.

### Detection of sIgA was performed using ELISA assay

Intestinal tissue was obtained by scraping 0.2 g of intestinal mucosa and washing three times with PBS solution. All procedures were performed under ice-cold conditions. Cell lysis buffer and protease inhibitor were mixed at a ratio of 100:1 (vol/vol) and vortexed. The washed intestinal mucosal tissue was placed in a 2.5 ml EP tube, 1 ml of the prepared solution was added and steel beads were added. The tissue was homogenized using an ultrasonic disintegrator and centrifuged at 3,000 rpm for 15 minutes to obtain the supernatant for further use. SIgA levels were detected by ELISA assay according to the detailed instructions of the kit (Jingmei, Jiangsu, China).

### Western blotting was used to detect IL-1β, IL-6, and IL-17 in the intestinal mucosal tissue

**Figure 1:**
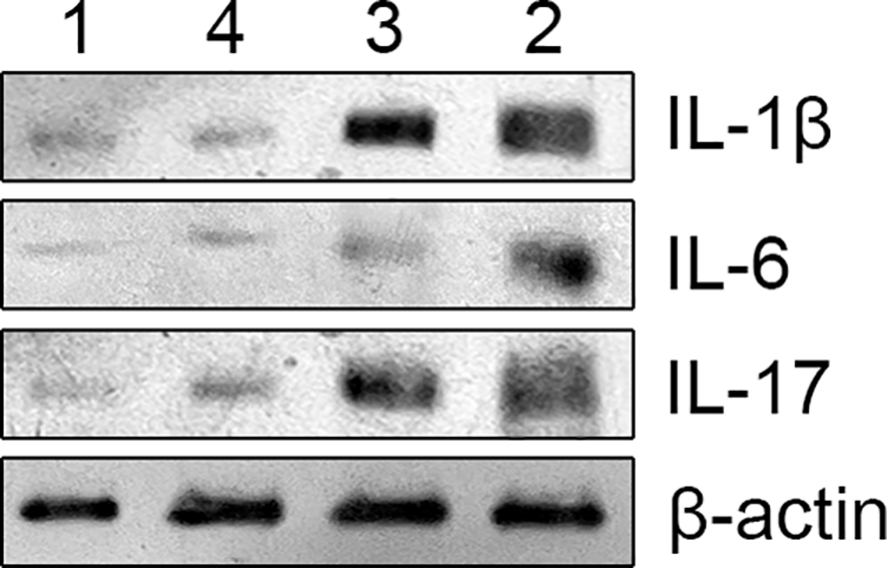
The above image is an electrophoretic image of protein blotting method for detecting cytokines, with different cytokines represented on the right and different groups marked on the top. group 1 (non-radiation group), group 2 (60 ± 2 days post-radiation), group 3 (75 ± 2 days post-radiation), group 4 (90 ± 2 days post-radiation).

The samples were diluted to the same concentration with lysis buffer and equal amounts of sample buffer were taken into test tubes containing 70 ug of protein. After heat treatment at 95-100°C and cooling on ice for 5 min, the samples were loaded onto the gel. Electrophoresis conditions included 20 minutes of constant voltage at 80V for the stacking gel and 80 minutes at 100V for the separating gel. The gel was removed and soaked in transfer buffer for 15 min. The filter paper and PVDF membrane were prepared and placed in the transfer buffer and deionised water respectively. The gel was then sandwiched between the filter paper, PVDF membrane and filter paper and the electrodes were placed on the layer. After removing air bubbles from each layer, the top electrode was placed on the sandwich material and a constant current of 200 mA was applied for 1 h. The PVDF membrane was blocked in 5% skimmed milk blocking solution for 1 hour at room temperature and the solution was discarded without washing. The membrane was incubated with an appropriate amount of primary antibody (IL-1β, CST, USA; IL-6, CST, USA; IL-17, abcam, UK)against beta-actin(IL-1β, CST, USA (Immunoway, USA) (1:4000) and blocking solution on a shaker (4°C, overnight). The membrane was washed four times with PBST for 5 minutes each time. A secondary antibody (horseradish peroxidase-conjugated antibody(Jackson, USA), dilution 1:5000) that binds to HRP was added to the membrane, which was incubated on a shaker at room temperature for 1-2 hours. The membrane was then thoroughly washed with PBST five times for 5 min each. The amount of developing solution was calculated as 0.1 mL/cm2, added to the PVDF membrane and incubated at room temperature for 1 min. The membrane was then wrapped in plastic foil (avoiding air bubbles as far as possible). The membrane was rapidly exposed to x-ray film in a dark room, developed and cleaned in an automatic film processor. Adjust the exposure time until the optimum band appears. **Figure 1** shows the electrophoretic bands of cytokines detected by Western blotting.

### Flow cytometry was used to detect CD4 and CD8

When the cell fusion rate in the culture bottle reaches 80%~90%, after removing the original culture medium, 0.25% trypsin is added to digest for 1-2 minutes, then an equal amount of culture medium containing serum is added to complete the digestion. The cells are suspended and centrifuged (using a pipette to aspirate the cells), then resuspended and transferred to a culture flask for further cultivation. Logarithmic growth phase cells were collected after digestion with 0.25% trypsin, washed twice with phosphate-buffered saline (PBS) (2000 rpm, 5 min centrifugation) and 5×10^5 cells were collected. Fluorescence-labelled lectin antibody was added, incubated at room temperature in the dark for 15 min, then centrifuged again at 1500 rpm for 5 min and the supernatant discarded. The cell pellets were washed twice with PBS and then 0.5 ml PBS was added to form a single cell suspension. Flow cytometry was used to detect cell surface antigen expression (Ex = 488 nm, Em = 530 nm, Becton Dickinson FACS Calibur, USA).

### 16s rRNA gene sequencing

#### Extraction and PCR amplification of genomic DNA

The genomic DNA of the sample is extracted by CTAB or SDS method, and then the purity and concentration of DNA are detected by agarose gel electrophoresis. Take an appropriate amount of sample DNA in a centrifuge tube, and dilute the sample to 1ng with sterile water/μ L. Using the diluted genomic DNA as a template and based on the selection of sequencing regions, specific primers with Barcode were used, using Phusion from New England Biolabs ^®^ High Fidelity PCR Master Mix with GC Buffer and efficient high-fidelity enzymes for PCR to ensure amplification efficiency and accuracy.

Primer corresponding area:

16S V4 primer (515F and 806R): identification of bacterial diversity;

18S V4 primer (528F and 706R): identification of eukaryotic microbial diversity;

ITS1 primer (ITS5-1737F and ITS2-2043R): identification of fungal diversity;

In addition, the amplification region also includes: 16S V3-V4/16S V4-V5/16SV5-V7; Archaea 16S V4-V5/Archaea 16S V8; 18S V9 and ITS2 zones.

#### Mixing and purification of PCR products

PCR products were detected by electrophoresis using agarose gel with 2% concentration; The qualified PCR products were purified by magnetic beads, quantified by enzyme label, and mixed in equal amounts according to the concentration of the PCR products. After full mixing, the PCR products were detected by 2% agarose gel electrophoresis. For the target strip, the gel recovery kit provided by Qiagen Company was used to recover the products.

#### Library construction and machine sequencing

Using TruSeq ^®^ The DNA PCR-Free Sample Preparation Kit kit was used for library construction. The constructed library was quantified by Qubit and Q-PCR, and after passing the library, NovaSeq6000 was used for machine sequencing.

### Statistical analysis

Statistical analysis was conducted using SPSS 26.0 software. Normal distribution and homoscedasticity measurement data are represented as mean ± standard deviation. Compare the non radiation group (Group 1) and different time points after radiation (Groups 2, 3, and 4) using independent sample t-tests. Single factor analysis of variance is used to compare groups at different time points after radiation. The median (quartile) is used for comparison between groups that do not meet the above criteria. If p<0.05, the difference is considered statistically significant. Significant differences are indicated: *p < 0.05; **p < 0.01; △p > 0.05.

## Results

### Changes in intestinal mucosal SIgA follow a certain pattern

The study revealed specific patterns in the changes of intestinal mucosal SIgA following radiation-induced injury. There was a decrease in SIgA content in the intestinal mucosa post-radiation, lower than that in the non-radiation group. While there was a gradual recovery over time, SIgA levels did not return to normal within 8-12 weeks. The comparison between the non-radiation group and different post-radiation time intervals utilized t-tests. For comparisons among various time intervals post-radiation, one-way ANOVA was applied. Detailed results are illustrated in the accompanying figure.

### CD4 and CD8 subgroups undergo changes

**Figure 2:**
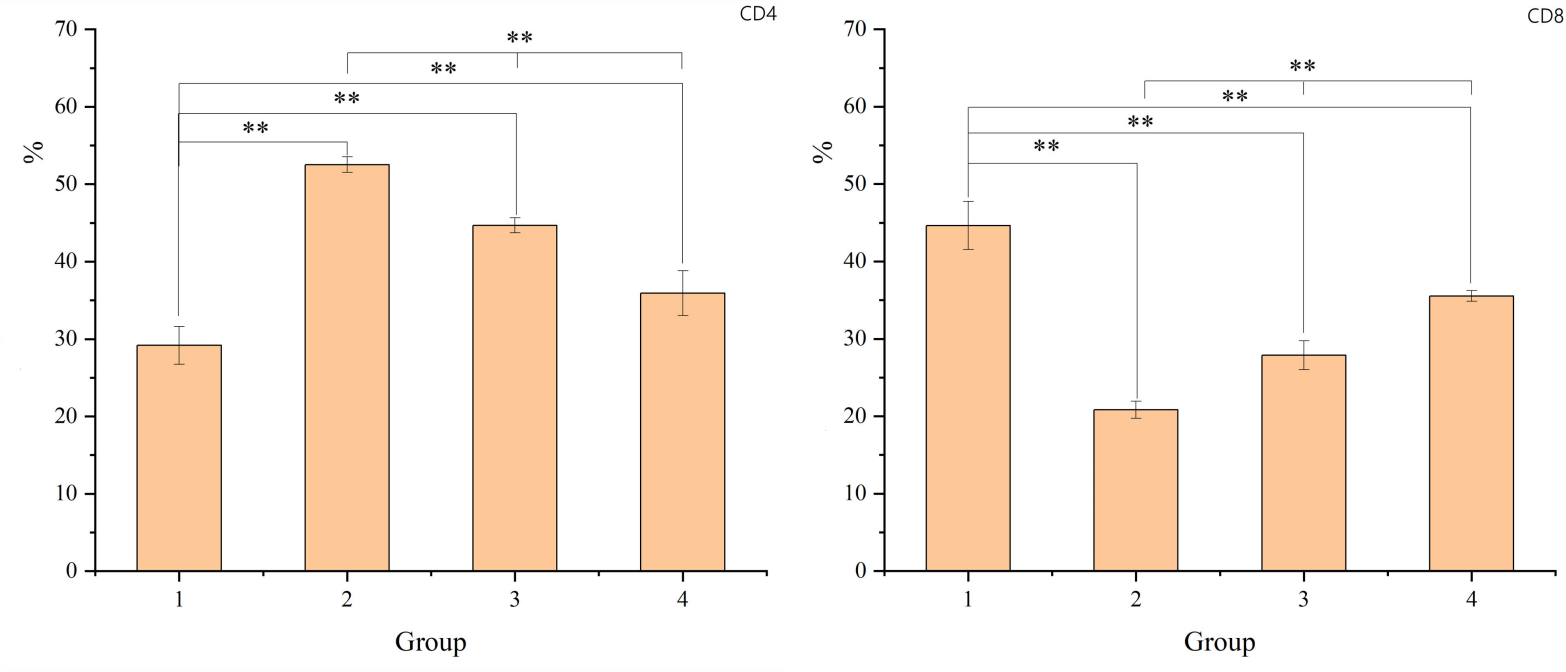
The figure shows the proportion of CD4 and CD8+ T lymphocytes. The X-axis represents the sample number, which corresponds to G1 (non-radiation group), G2 (60 ± 2 days post-radiation), G3 (75 ± 2 days post-radiation), and G4 (90 ± 2 days post-radiation). The Y-axis represents the percentage of CD4 or CD8 lymphocytes. The mean, standard deviation and P-values for intergroup comparisons are shown in the table below. Significant differences are indicated: *p < 0.05; **p < 0.01; △p > 0.05.

Similarly, the study observed changes in the CD4 and CD8 lymphocyte subgroups. T-tests were used to compare the ratios of CD4 and CD8 subgroups between group 1 (non-radiation) and groups 2, 3, and 4 (post-radiation). One-way ANOVA was employed for comparisons among groups 2, 3, and 4. Post-radiation, there was an increase in the proportion of CD4+ T lymphocytes and a decrease in CD8+ T lymphocytes. Over time, these indices showed a gradual return to the levels observed in the non-radiation group. The results, including mean ± standard deviation and P-values, are detailed in the figure provided. The result is shown in **Figure 2**.

### Intestinal mucosal barrier damage leads to changes in cytokine levels

**Figure 3:**
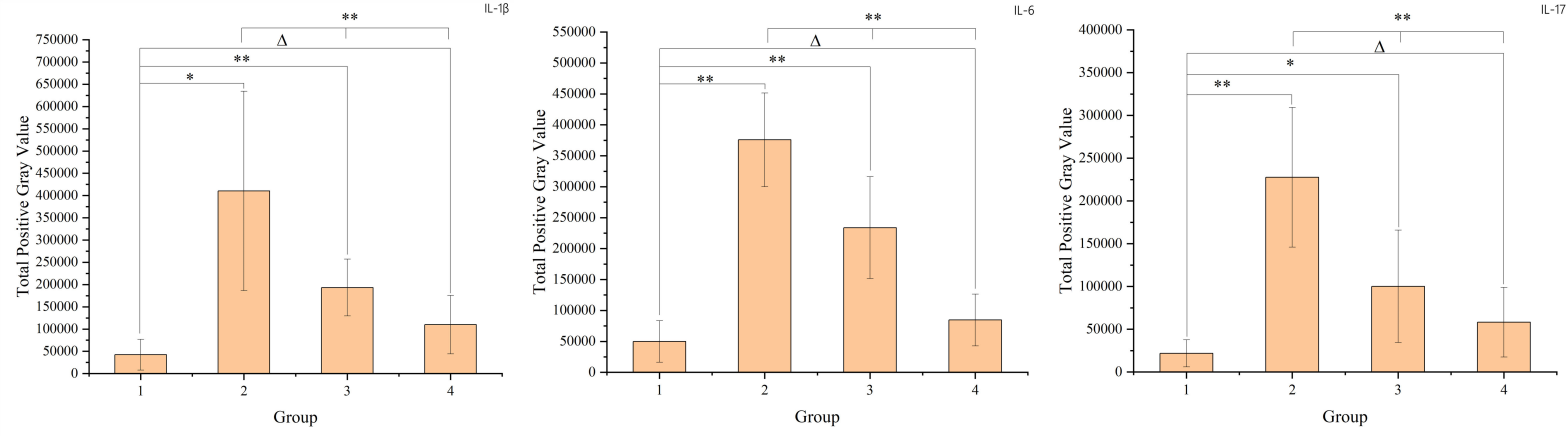
The figure shows the cytokine levels in intestinal mucosal tissues of each sample. Panel a represents IL-1β, panel b represents IL-6, and panel c represents IL-17. The X-axis represents the sample number, where samples 1-6 belong to group 1 (non-radiation group), samples 7-12 belong to group 2 (60 ± 2 days post-radiation), samples 13-18 belong to group 3 (75 ± 2 days post-radiation), and samples 19-24 belong to group 4 (90 ± 2 days post-radiation). The Y-axis represents the total grayscale value of the WB detection band for each cytokine. The mean, standard deviation and P-values for intergroup comparisons are shown in the figure below.

The study found that radiation-induced injury led to increased levels of cytokines such as IL-1β, IL-6, and IL-17 in the intestinal mucosa. Over time, these cytokine levels gradually returned to those observed in the non-radiation group. To compare the non-radiation group (G1) with the different post-radiation time intervals (G2, G3, G4), t-tests were used. For comparisons among the different time intervals post-radiation, one-way ANOVA was employed. These results, including the specific changes in cytokine levels over time, are presented in the figure below. The result is shown in **Figure 3**.

### Radiation-induced intestinal mucosal barrier damage can lead to changes in oral microbiota

**Figure 4:**
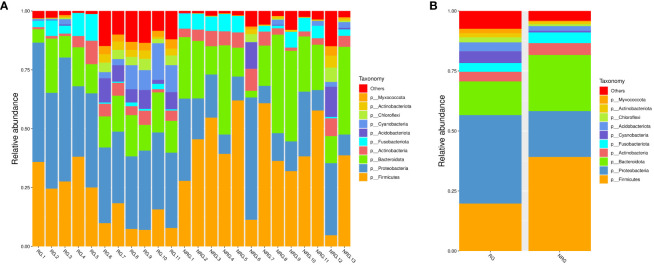
Based on the species annotation results, the top 10 species with the highest relative abundance at the phylum level were selected for each sample to generate a stacked bar chart of the relative abundance of species, in order to visually observe the species with higher relative abundance and their proportions at different classification levels in each sample. **(A)** a stacked bar chart of the relative abundance of species at the phylum level based on OTU, where the x-axis represents the sample name, the y-axis (Relative Abundance) represents the relative abundance, and “Others” represents the sum of the relative abundance of all other phyla besides these 10 phyla in the figure. **(B)** a stacked bar chart of the relative abundance of species at the phylum level based on OTU for different groups, where the x-axis represents the grouping, the y-axis (Relative Abundance) represents the relative abundance, and “Others” represents the sum of the relative abundance of all other phyla besides these 10 phyla in the figure. ANOSIM analysis showed significant differences in similarity between groups (R = 0.1989, p=0.021).

**Figure 5:**
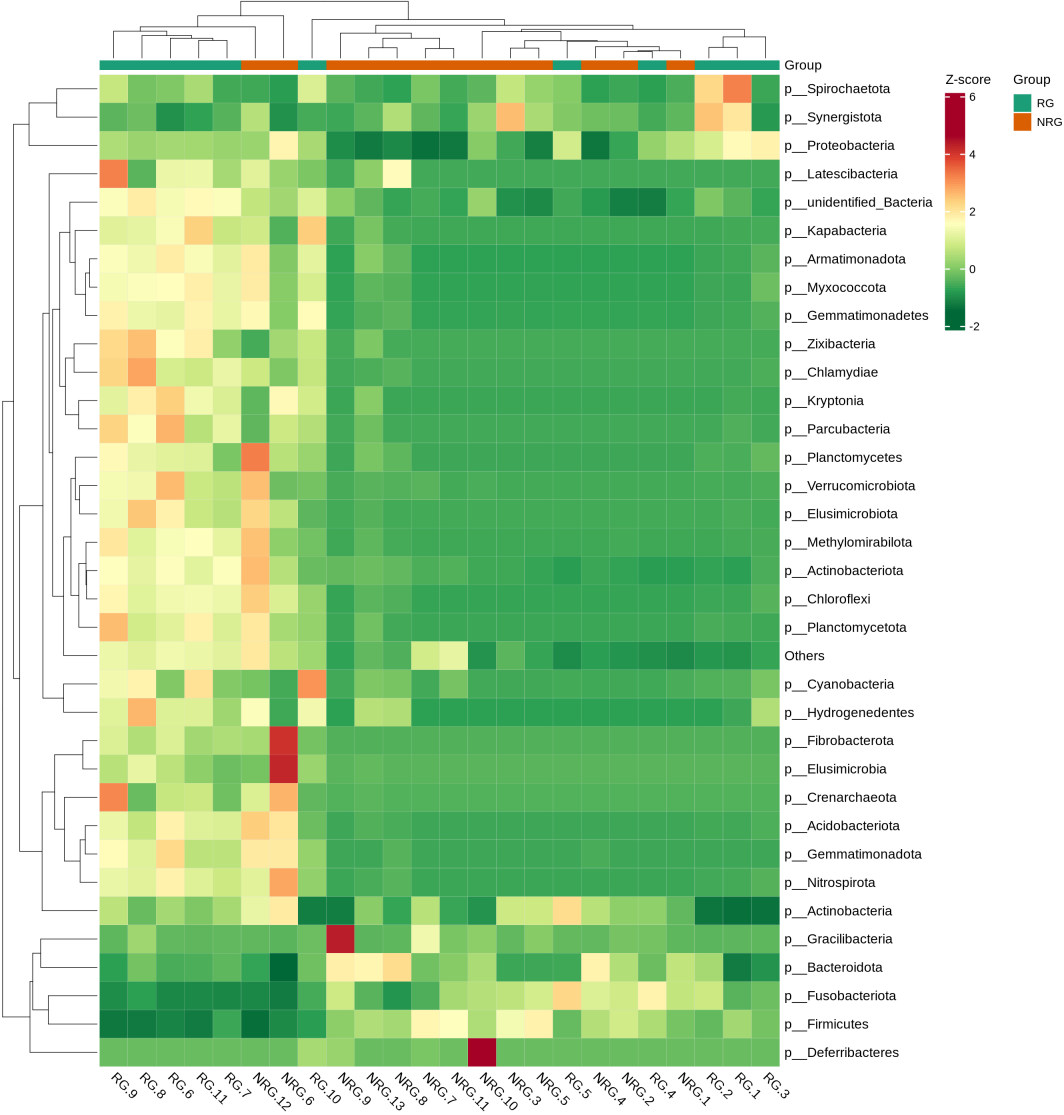
A heatmap of quantitative data based on OTU and a Metastats significance difference identification diagram. The top 35 microbial classifications with the highest quantitative values in all samples were selected. Based on their quantitative information in each sample, clustering was performed at both the species and sample levels to create a heatmap, which facilitates the identification of which species are more abundant or less abundant in which samples and allows for the identification of clustering relationships between samples. The vertical axis represents sample information and the horizontal axis represents species classification information. The clustering tree in the figure represents species clustering. The values in the heatmap correspond to Z-Score standardized relative quantitative data, and the colors on the right side represent the Metastats significance of microbial differences in the corresponding group.

Saliva samples from 24 rectal cancer patients were collected and divided into two groups: the radiotherapy group (RG, n=11) and the non-radiotherapy group (NRG, n=13). Through 16S rRNA gene sequencing, differences in the top ten microbiota at the phylum level were observed between the two groups, including Firmicutes, Proteobacteria, Bacteroidota, and Cyanobacteria (Anosim analysis R=0.1989, p=0.021). **Figure 4** displays a stacked bar chart illustrating the relative abundance of microbial species at the phylum level based on Operational Taxonomic Units (OTUs). **Figure 5** presents a heatmap of quantitative data based on OTUs and a Metastats significance difference identification diagram. Significant differences were noted between the two groups in Proteobacteria, Cyanobacteria, and Firmicutes (P<0.01), and in 12 other bacterial groups, including Spirochaetota and Myxococcota (P<0.05).

**Figure 6:**
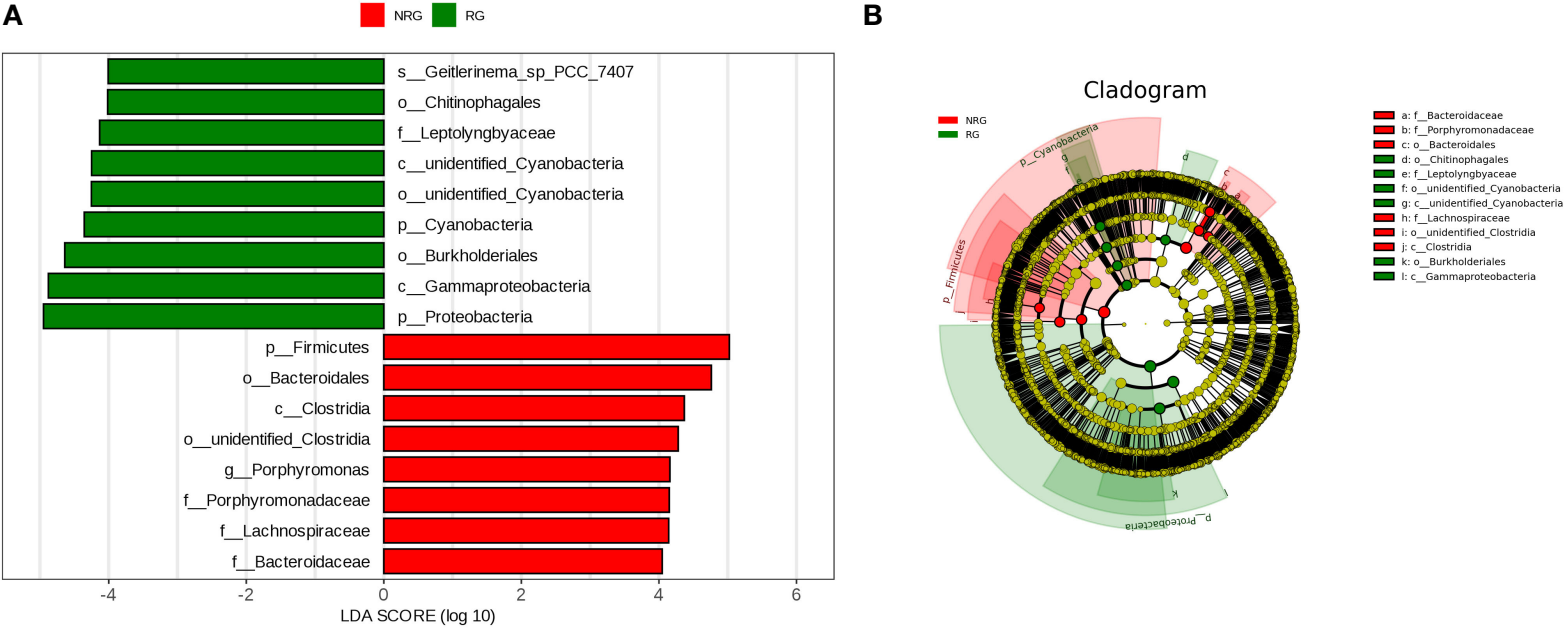
Panel **(A)** is a LDA value distribution histogram based on OTU. The histogram shows the species with LDA Score greater than the set value (default set to 4), which are biomarkers with statistical differences between groups. The histogram displays the significantly different species in abundance between different groups, and the length of the bar represents the magnitude of the impact of the different species (i.e. LDA Score). Panel **(B)** is an evolutionary branching diagram based on OTU, where the circles radiating from the inside out represent the classification levels from phylum to genus (or species). Each small circle at each classification level represents a classification at that level, and the diameter of the small circle is proportional to the relative abundance. The coloring principle is as follows: species with no significant differences are uniformly colored yellow, biomarkers of different species follow the group coloring, red nodes represent microbial groups that play an important role in the red group, and green nodes represent microbial groups that play an important role in the green group. The species names represented by English letters in the figure are displayed in the legend on the right side.

The LEfSe (LDA Effect Size) analysis tool, which emphasizes statistical significance and biological relevance, identified biomarkers with statistical differences between the groups. The statistical results of LEfSe include a distribution histogram of LDA values, an evolutionary branching diagram (phylogenetic distribution), and the comparative abundance of biomarkers with statistical differences between groups. **Figure 6A** depicts the LDA value distribution histogram based on OTUs, and **Figure 6B** presents an evolutionary branching diagram based on OTUs. The analysis revealed that in the radiotherapy group, 9 bacterial groups, including Proteobacteria and Burkholderia, showed significant differences, while in the untreated group, 8 bacterial groups, such as Firmicutes, Bacteroidetes, and Clostridium, exhibited notable differences.

## Discussion

Colorectal cancer (CRC), the third most common malignant tumor, often requires neoadjuvant therapy, particularly preoperative radiotherapy, for advanced stages. Radiotherapy is crucial in reducing tumor growth, size and metastasis (Bullman et al., 2017). However, it also carries risks, in particular damage to the intestinal mucosal barrier, which may be associated with perioperative complications. The intestinal microbiota, an important part of this biological barrier, is altered in CRC patients, with consequences for the corresponding oral microbiota.

Is the primary antibody type in the intestinal mucosa and acts as the first line of defense against pathogens. It prevents pathogen adhesion and invasion, promotes colonization by commensal bacteria and maintains immune homeostasis (Sutherland and Fagarasan, 2012; Pietrzak et al., 2020). Thus, SIgA levels may reflect the state of the intestinal mucosal immune barrier. Radiation therapy can affect this barrier, increasing intestinal permeability and compromising barrier integrity (Nejdfors et al., 2000). The intestinal epithelium and lamina propria, where CD8 T lymphocytes predominate (approximately 80%), are key sites for assessing changes in intestinal immune function (Liu et al., 2021). For example, ischaemia-reperfusion-induced damage to the intestinal mucosal barrier has been associated with an increase in CD8+ T lymphocytes (Wang et al., 2023). Interleukin serves as an early inflammatory mediator, stimulating inflammatory responses, cell proliferation and apoptosis. In particular, IL-1β can activate intestinal epithelial and immune cells to produce inflammatory factors such as TNF-α, IL-6 and IL-8, thereby triggering intestinal inflammatory responses. It can also increase the permeability of intestinal tight junctions (TJs), facilitating endothelial cells to produce vascular endothelial growth factor and create an inflammatory microenvironment conducive to tumor progression (Al-Sadi et al., 2010; Rawat et al., 2020; Karpiński, 2019). IL-17, another important cytokine, promotes inflammatory cell infiltration and the release of inflammatory factors. Rovedatti et al. found increased IL-17 expression in the inflamed mucosa of patients with inflammatory bowel disease (Rovedatti et al., 2009).

**Figure 7:**
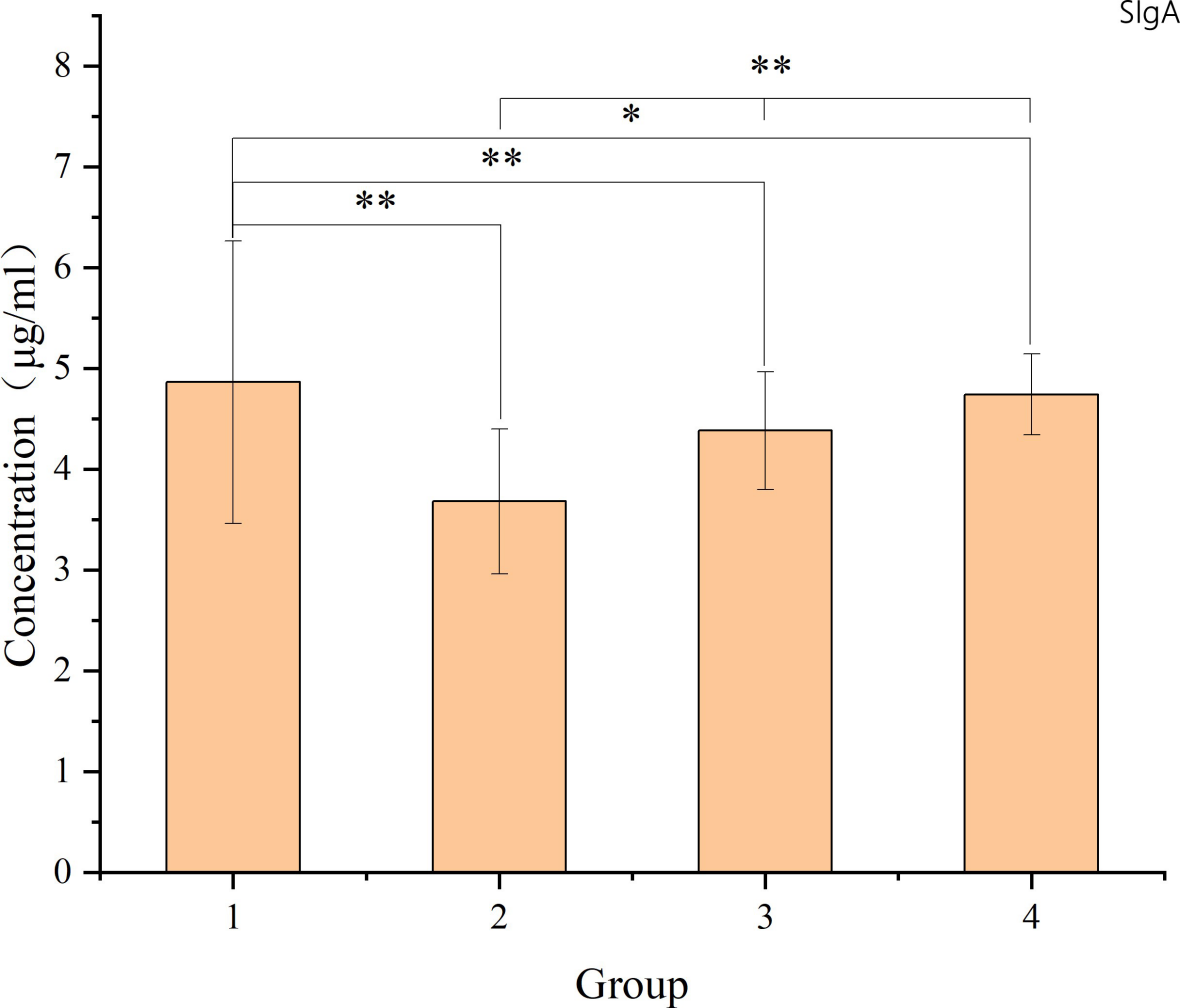
The figure shows the SIgA levels in intestinal mucosal tissues of each sample. The X-axis represents the sample number, where samples 1-9 belong to group 1 (non-radiation group), samples 10-17 belong to group 2 (60 ± 2 days post-radiation), samples 18-27 belong to group 3 (75 ± 2 days post-radiation), and samples 28-34 belong to group 4 (90 ± 2 days post-radiation). The Y-axis represents the SIgA content (μg/ml). The mean, standard deviation and P-values for intergroup comparisons are shown in the figure below. Significant differences are indicated: *p < 0.05; **p < 0.01; △p > 0.05.

As shown in **Figure 7**, the levels of SIgA in the intestinal mucosal barrier 8-12 weeks after radiotherapy were significantly lower than in patients who did not receive radiotherapy (p<0.05). There was a gradual increase in SIgA levels from 8-12 weeks, suggesting that radiation-induced damage to the intestinal mucosal barrier leads to impaired immune barrier function. However, even 8-12 weeks after radiotherapy, the intestinal mucosal immune barrier was still recovering and had not fully returned to normal levels.

Our study also observed a significant decrease in the proportion of CD8+ T lymphocytes in the intestinal mucosa after radiotherapy, while the proportion of CD4+ T lymphocytes increased relatively. This change suggests that radiotherapy damages the function of the intestinal mucosal immune barrier. However, there was a gradual recovery to near-normal levels 8-12 weeks after radiotherapy.

The increase in cytokines such as IL-1β, IL-6 and IL-17 in the intestinal mucosa after radiotherapy indicates the onset of radiation enteritis. The subsequent decrease in cytokine levels 8-12 weeks after radiotherapy suggests that the intestinal mucosal barrier is in a recovery phase during this period. However, the barrier function had not fully returned to normal levels even 12 weeks after radiotherapy.

There are several differences in the salivary microbiota between patients with rectal cancer after radiotherapy and those without radiotherapy. Changes in the gut microbiota can induce intestinal inflammation and affect the expression of tight junction proteins, thereby increasing intestinal permeability (Suzuki, 2013). The interplay between oral and gut microbiota appears to influence the efficacy and prognosis of radiotherapy for rectal cancer (Dong et al., 2021). Radiation-induced damage to the intestinal mucosal barrier may lead to systemic changes in both the gut and oral microbiota, potentially influencing the occurrence and prognosis of perioperative complications.

In healthy individuals, the composition of the gut microbiota is relatively stable, consisting predominantly of Bacteroidetes and Firmicutes (Mirpuri et al., 2014; Jandhyala et al., 2015). As shown in **Figures 4**–**6**, the oral microbiota of patients in the non-radiotherapy group is similar to that of healthy individuals in terms of gut microbiota. Conversely, the saliva of patients in the radiotherapy group showed significant changes, indicating an altered microbial abundance.

The use of probiotics to regulate the gut microbiota has been suggested as an effective strategy to reduce complications in surgical patients (Bajramagic et al., 2019). The 2021 study by Dong et al. further strengthens the link between oral microbiota and treatment outcomes, as shown by 16S rRNA sequencing results. Their research in a CRC mouse model showed that changes in the oral microbiota can alter the composition of intestinal bacteria within tumours. This interaction between oral and gut microbiota may significantly influence the efficacy and prognosis of CRC radiotherapy. The study highlights the complex relationship between oral microbiota, the intestinal mucosal barrier and gastrointestinal tumors (Dong et al., 2021).

In conclusion, neoadjuvant radiotherapy is a standard treatment to reduce postoperative recurrence and metastasis in patients with stage II-III colorectal cancer (Wo et al., 2021). However, this therapy can compromise the intestinal mucosal barrier, leading to increased local reactions and decreased immune function, which may contribute to perioperative complications. The timing of surgery after radiotherapy is critical: too early and problems such as tissue oedema and insufficient tumor shrinkage from radiochemotherapy can complicate surgery; too late and there’s a risk of tumor progression, spread and reduced compliance with neoadjuvant treatment, missing the optimal surgical window. Ming Yii Huang et al. have summarized advances in neoadjuvant radiotherapy and chemotherapy for locally advanced rectal cancer, including the optimal interval between therapy and surgery (Huang et al., 2020). Current guidelines typically recommend surgery 8 to 12 weeks after completion of radiotherapy (Glynne-Jones et al., 2017). During this time, the intestinal mucosal barrier function gradually recovers. Delaying surgery slightly within the 8-12 week window may reduce perioperative complications and improve patient recovery.

However, in clinical practice, the complexity of each patient’s condition requires personalized treatment planning. Following radiotherapy, rectal cancer patients may experience changes in the oral microbiota due to damage to the intestinal mucosal barrier. Integrating metabolomics to test for different metabolites could provide a novel, non-invasive method to monitor the recovery of gut mucosal barrier function after radiotherapy. This approach also opens avenues for the development of new microbial modulation agents and probiotics to mitigate post-operative complications of colorectal cancer and counteract the adverse effects of radiotherapy-induced damage.

In colorectal cancer, oncological aspects often receive more attention, while studies focusing on the intestinal mucosal barrier are less common. This study contributes to this under-explored area by examining post-operative intestinal mucosal tissue and refining the timeframe after radiotherapy as outlined in guidelines. It aims to understand the repair mechanisms of the intestinal mucosal barrier and reduce complications arising from radiotherapy-induced damage. The relationship between oral microbiota and systemic diseases has gained interest in recent years. However, the literature on the oral microbiota after radiotherapy for rectal cancer remains scarce. This study addresses this gap by analyzing changes in the salivary microbiota of rectal cancer patients before and after radiotherapy, shedding light on microbiota changes. It aims to lay the groundwork for identifying suitable probiotics or immunomodulators to alleviate perioperative complications and improve the prognosis of CRC patients following radiotherapy. Despite its contributions, the study acknowledges certain limitations, such as the relatively small sample size and the single-centre nature of the study. Future research plans include increasing the sample size and conducting multicenter studies, provided the necessary conditions are met.

## Conclusions

This research reveals that rectal cancer patients who undergo radiotherapy and those who undergo abdominal surgery without radiotherapy exhibit changes in their oral microbiota. These findings suggest potential for non-invasive monitoring of intestinal mucosal barrier recovery and the development of probiotics to mitigate perioperative complications caused by radiotherapy-induced damage. It’s important to note that while the intestinal mucosal barrier function gradually recovers, it does not fully return to normal levels in patients undergoing surgery 8-12 weeks after radiotherapy.

## Data availability statement

The authors acknowledge that the data presented in this study must be deposited and made publicly available in an acceptable repository, prior to publication. Frontiers cannot accept a article that does not adhere to our open data policies.

## Ethics statement

The studies involving humans were approved by Medical Ethics Committee of Qingdao University Affiliated Hospital. The studies were conducted in accordance with the local legislation and institutional requirements. The participants provided their written informed consent to participate in this study.

## Author contributions

KW: Formal analysis, Investigation, Methodology, Writing – original draft, Writing – review & editing. JZ: Data curation, Investigation, Writing – original draft, Writing – review & editing. YZ: Resources, Writing – review & editing. JX: Formal analysis, Writing – review & editing. HW: Methodology, Writing – review & editing. XT: Methodology, Writing – review & editing. XJ: Data curation, Writing – review & editing. HJ: Funding acquisition, Methodology, Resources, Writing – review & editing.

## References

[B1] Al-Sadi R. Ye D. Said H. M. Ma T. Y. (2010). IL-1beta-induced increase in intestinal epithelial tight junction permeability is mediated by MEKK-1 activation of canonical NF-kappaB pathway. Am. J. Pathol. 177 (5), 2310–2322. doi: 10.2353/ajpath.2010.100371 21048223 PMC2966790

[B2] Bajramagic S. Hodzic E. Mulabdic A. Holjan S. Smajlovic S. V. Rovcanin A. (2019). Usage of probiotics and its clinical significance at surgically treated patients sufferig from colorectal carcinoma. Med. Arch. 73 (5), 316–320. doi: 10.5455/medarh.2019.73.316-320 31819304 PMC6885229

[B3] Bullman S. Pedamallu C. S. Sicinska E. Clancy T. E. Zhang X. Cai D.. (2017). Analysis of fusobacterium persistence and antibiotic response in colorectal cancer. Science 358 (6369), 1443–1448. doi: 10.1126/science.aal5240 29170280 PMC5823247

[B4] Chen Y. Cui W. Li X. Yang H. (2021). Interaction between commensal bacteria, immune response and the intestinal barrier in inflammatory bowel disease. Front. Immunol. 12, 761981. doi: 10.3389/fimmu.2021.761981 34858414 PMC8632219

[B5] Dong J. Li Y. Xiao H. Zhang S. Wang B. Wang H.. (2021). Oral microbiota affects the efficacy and prognosis of radiotherapy for colorectal cancer in mouse models. Cell Rep. 37 (4), 109886. doi: 10.1016/j.celrep.2021.109886 34706245

[B6] Fan J. Lin B. Fan M. Niu T. Gao F. Tan B.. (2022). Research progress on the mechanism of radiation enteritis. Front. Oncol. 12, 888962. doi: 10.3389/fonc.2022.888962 36132154 PMC9483210

[B7] Flemer B. Warren R. D. Barrett M. P. Cisek K. Das A. Jeffery I. B.. (2018). The oral microbiota in colorectal cancer is distinctive and predictive. Gut 67 (8), 1454–1463. doi: 10.1136/gutjnl-2017-314814 28988196 PMC6204958

[B8] Garrett W. S. (2015). Cancer and the microbiota. Science 348 (6230), 80–86. 25838377 10.1126/science.aaa4972 PMC5535753

[B9] Glynne-Jones R. Wyrwicz L. Tiret E. Brown G. Rödel C. Cervantes A.. (2017). Rectal cancer: ESMO clinical practice guidelines for diagnosis, treatment and follow-up. Ann. Oncol. 28 (suppl_4), iv22–iv40. doi: 10.1093/annonc/mdz400 28881920

[B10] Huang M. Y. Huang C. W. Wang J. Y. (2020). Surgical treatment following neoadjuvant chemoradiotherapy in locally advanced rectal cancer. Kaohsiung J. Med. Sci. 36 (3), 152–159. doi: 10.1002/kjm2.12161 31814296 PMC11896401

[B11] Jandhyala S. M. Talukdar R. Subramanyam C. Vuyyuru H. Sasikala M. Nageshwar Reddy D. (2015). Role of the normal gut microbiota. World J. Gastroenterol. 21 (29), 8787–8803. doi: 10.3748/wjg.v21.i29.8787 26269668 PMC4528021

[B12] Karpiński T. M. (2019). Role of oral microbiota in cancer development. Microorganisms 7 (1). doi: 10.3390/microorganisms7010020 PMC6352272 30642137

[B13] Kwak S. Y. Jang W. I. Park S. Cho S. S. Lee S. B. Kim M. J.. (2021). Metallothionein 2 activation by pravastatin reinforces epithelial integrity and ameliorates radiation-induced enteropathy. EBioMedicine 73, 103641. doi: 10.1016/j.ebiom.2021.103641 34688032 PMC8546423

[B14] Lin Y. Xia P. Cao F. Zhang C. Yang Y. Jiang H.. (2023). Protective effects of activated vitamin D receptor on radiation-induced intestinal injury. J. Cell Mol. Med. 27 (2), 246–258. doi: 10.1111/jcmm.17645 36579449 PMC9843524

[B15] Liu Z. Mi F. Han M. Tian M. Deng L. Meng N.. (2021). Bone marrow-derived mesenchymal stem cells inhibit CD8(+) T cell immune responses *via* PD-1/PD-L1 pathway in multiple myeloma. Clin. Exp. Immunol. 205 (1), 53–62. doi: 10.1111/cei.13594 33735518 PMC8209616

[B16] MacNaughton W. K. (2000). Review article: new insights into the pathogenesis of radiation-induced intestinal dysfunction. Aliment Pharmacol. Ther. 14 (5), 523–528. doi: 10.1046/j.1365-2036.2000.00745.x 10792113

[B17] Mirpuri J. Raetz M. Sturge C. R. Wilhelm C. L. Benson A. Savani R. C.. (2014). Proteobacteria-specific IgA regulates maturation of the intestinal microbiota. Gut Microbes 5 (1), 28–39. doi: 10.4161/gmic.26489 24637807 PMC4049932

[B18] Nejdfors P. Ekelund M. Weström B. R. Willén R. Jeppsson B. (2000). Intestinal permeability in humans is increased after radiation therapy. Dis. Colon Rectum 43 (11), 1582–1587. doi: 10.1007/BF02236743 11089597

[B19] Pietrzak B. Tomela K. Olejnik-Schmidt A. Mackiewicz A. Schmidt M. (2020). Secretory igA in intestinal mucosal secretions as an adaptive barrier against microbial cells. Int. J. Mol. Sci. 21 (23). doi: 10.3390/ijms21239254 PMC7731431 33291586

[B20] Rawat M. Nighot M. Al-Sadi R. Gupta Y. Viszwapriya D. Yochum G.. (2020). IL1B increases intestinal tight junction permeability by up-regulation of MIR200C-3p, which degrades occludin mRNA. Gastroenterology 159 (4), 1375–1389. doi: 10.1053/j.gastro.2020.06.038 32569770 PMC11752806

[B21] Rovedatti L. Kudo T. Biancheri P. Sarra M. Knowles C. H. Rampton D. S.. (2009). Differential regulation of interleukin 17 and interferon gamma production in inflammatory bowel disease. Gut 58 (12), 1629–1636. doi: 10.1136/gut.2009.182170 19740775

[B22] Sémont A. François S. Mouiseddine M. François A. Saché A. Frick J.. (2006). Mesenchymal stem cells increase self-renewal of small intestinal epithelium and accelerate structural recovery after radiation injury. Adv. Exp. Med. Biol. 585, 19–30. doi: 10.1007/978-0-387-34133-0_2 17120774

[B23] Sutherland D. B. Fagarasan S. (2012). IgA synthesis: a form of functional immune adaptation extending beyond gut. Curr. Opin. Immunol. 24 (3), 261–268. doi: 10.1016/j.coi.2012.03.005 22503962

[B24] Suzuki T. (2013). Regulation of intestinal epithelial permeability by tight junctions. Cell Mol. Life Sci. 70 (4), 631–659. doi: 10.1007/s00018-012-1070-x 22782113 PMC11113843

[B25] Touchefeu Y. Montassier E. Nieman K. Gastinne T. Potel G. Bruley des Varannes S.. (2014). Systematic review: the role of the gut microbiota in chemotherapy- or radiation-induced gastrointestinal mucositis - current evidence and potential clinical applications. Aliment Pharmacol. Ther. 40 (5), 409–421. doi: 10.1111/apt.12878 25040088

[B26] Wang H. Wang K. Liu B. Bian X. Tan X. Jiang H. (2023). The efficacy of bone marrow mesenchymal stem cells on rat intestinal immune-function injured by ischemia/reperfusion. Heliyon 9 (5), e15585. doi: 10.1016/j.heliyon.2023.e15585 37131448 PMC10149202

[B27] Wo J. Y. Anker C. J. Ashman J. B. Bhadkamkar N. A. Bradfield L. Chang D. T.. (2021). Radiation therapy for rectal cancer: Executive summary of an ASTRO clinical practice guideline. Pract. Radiat. Oncol. 11 (1), 13–25. doi: 10.1016/j.prro.2020.08.004 33097436

